# Author Correction: Boosting output performance of sliding mode triboelectric nanogenerator by charge space-accumulation effect

**DOI:** 10.1038/s41467-020-18578-3

**Published:** 2020-09-09

**Authors:** Wencong He, Wenlin Liu, Jie Chen, Zhao Wang, Yike Liu, Xianjie Pu, Hongmei Yang, Qian Tang, Huake Yang, Hengyu Guo, Chenguo Hu

**Affiliations:** Department of Applied Physics, State Key Laboratory of Power Transmission Equipment & System Security and New Technology, Chongqing University, 400044 Chongqing, P.R. China

**Keywords:** Devices for energy harvesting, Electrical and electronic engineering, Applied physics

Correction to: *Nature Communications* 10.1038/s41467-020-18086-4, published online 26 August 2020.

The original version of this Article contained an error in Fig. 1j, where the correct Y-axis unit is “μC” instead of “mC”.

This has been corrected in both the PDF and HTML versions of the Article.

