# Supplementary material for: Boosting output performance of sliding mode triboelectric nanogenerator by charge space-accumulation effect
Source: Nat Commun. 2020 Aug 26;11:4277. doi: 10.1038/s41467-020-18086-4 (PMC7450049; doi:10.1038/s41467-020-18086-4)
Supplement: Supplementary file 1 — Supplementary Information [file 41467_2020_18086_MOESM1_ESM.pdf]

Supplementary Information

Boosting Output Performance of Sliding Mode Triboelectric Nanogenerator by Charge Space-accumulation Effect

He et al.

Supplementary Figures

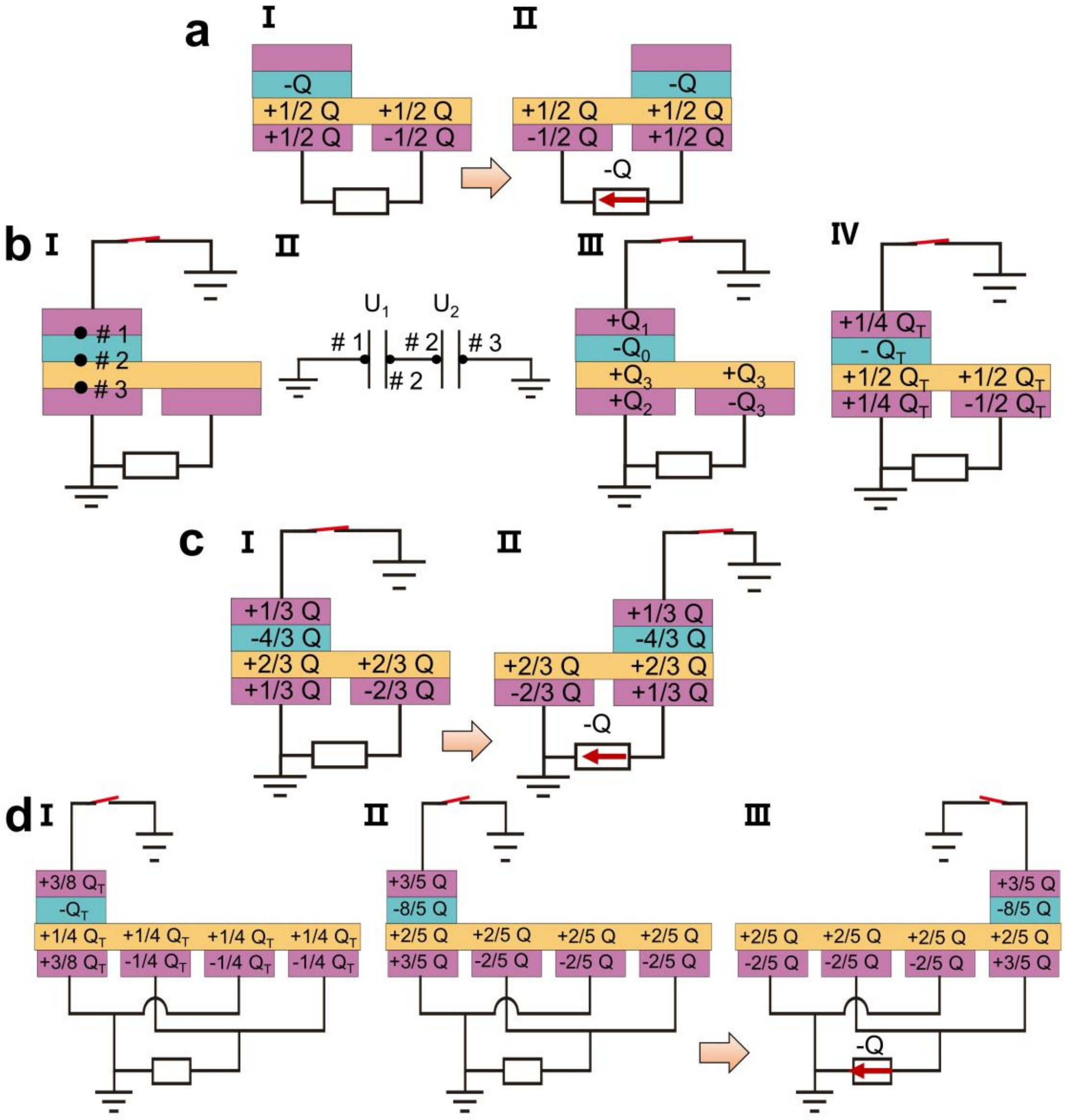

Supplementary Figure 1 | Working principle of traditional sliding TENG. a Working mechanism of the normal S-TENG. **b** Charge distribution of the S-TENG with the screening electrode. **c** Working mechanism of the S-TENG with the screening electrode when charge density of PTFE reached saturation again. **d** Charge distribution and working mechanism of the normal S-TENG with more electrode pairs.

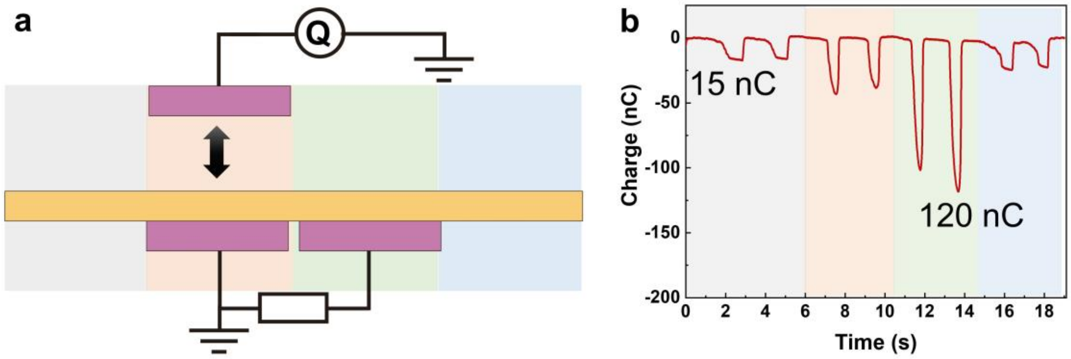

Supplementary Figure 2 | Charge dissipating on the surface of extra blank-tribo-area. **a** Schematic diagram of measurement of the remnant charges. **b** The remained charges in different areas of the CSA-S-TENG after several regular working cycles.

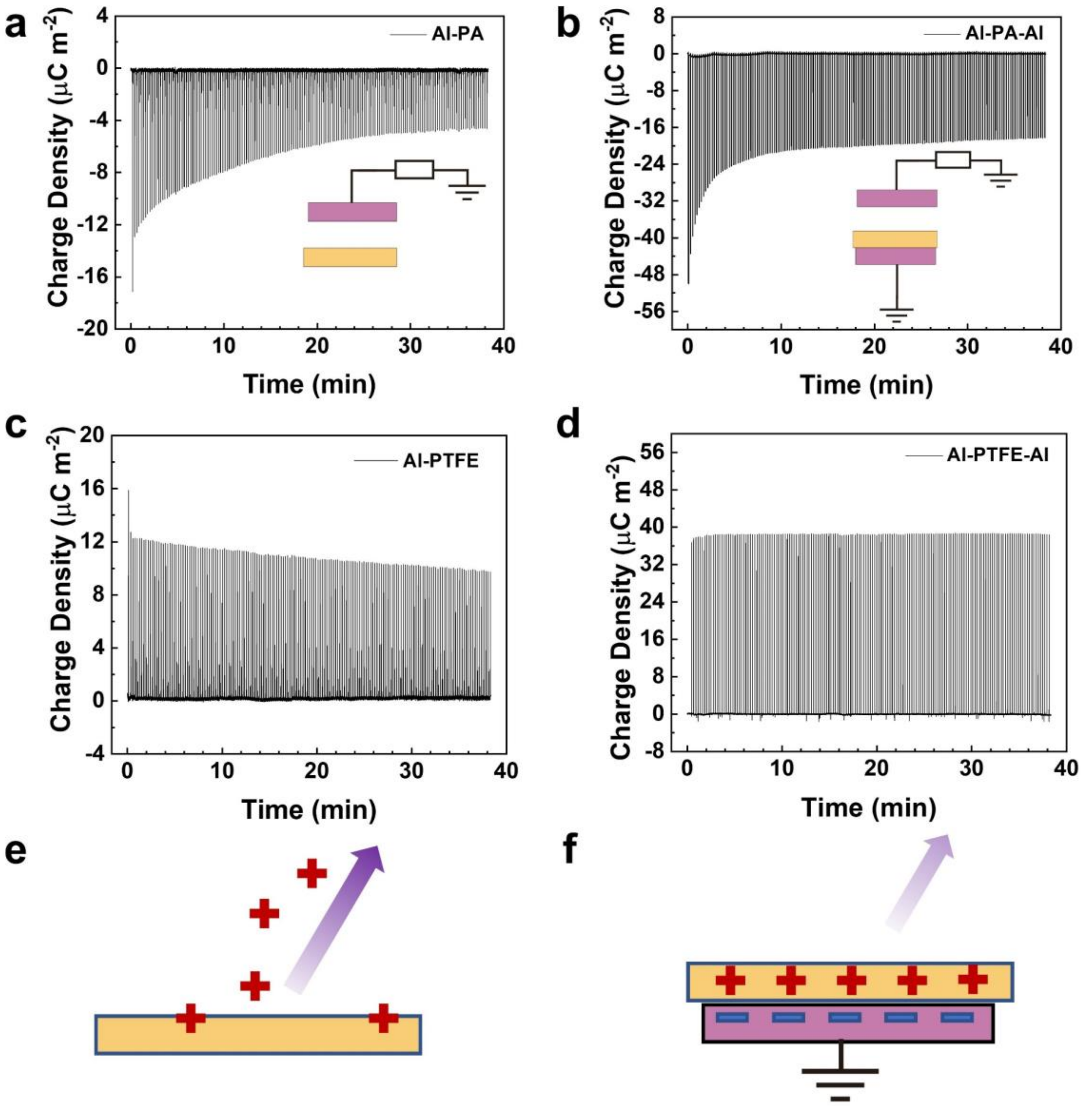

Supplementary Figure 3 | The ability of PA and PTFE to maintain surface charges. a Change of charge density under Al-PA single-electrode mode CS-TENG. **b** Change of charge density under Al-PA-Al double-electrode mode CS-TENG. **c** Change of charge density under Al-PTFE single-electrode mode CS-TENG. **d** Change of charge density under Al-PTFE-Al double electrodes mode CS-TENG. Sufficient charges were injected into the surface of the PA thin film and PTFE thin film before measurement. **e** The charges on surface of the thin film without electrode underneath are easy to dissipate into air. **f** The charges on surface of the thin film with electrode underneath, shielded by the charges on the bottom electrode, are hard to dissipate into air.

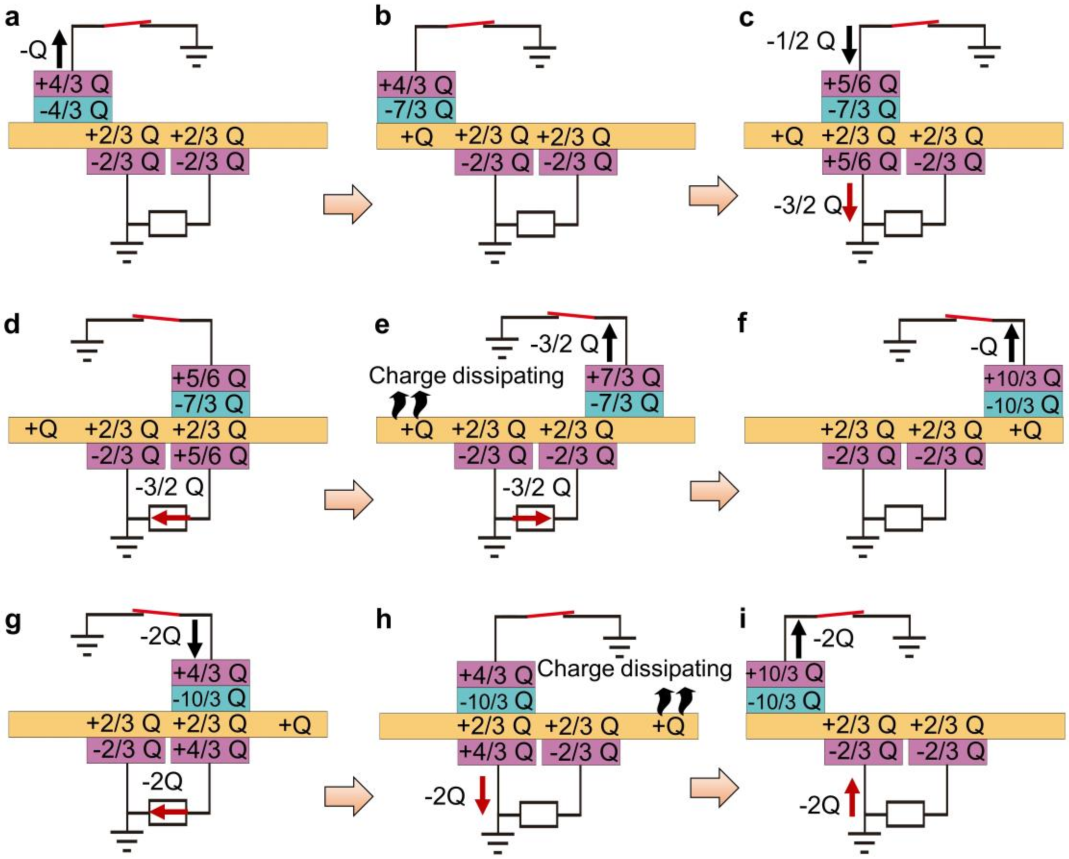

Supplementary Figure 4 | Working mechanism of the first cycle of the CSA-S-TENG.

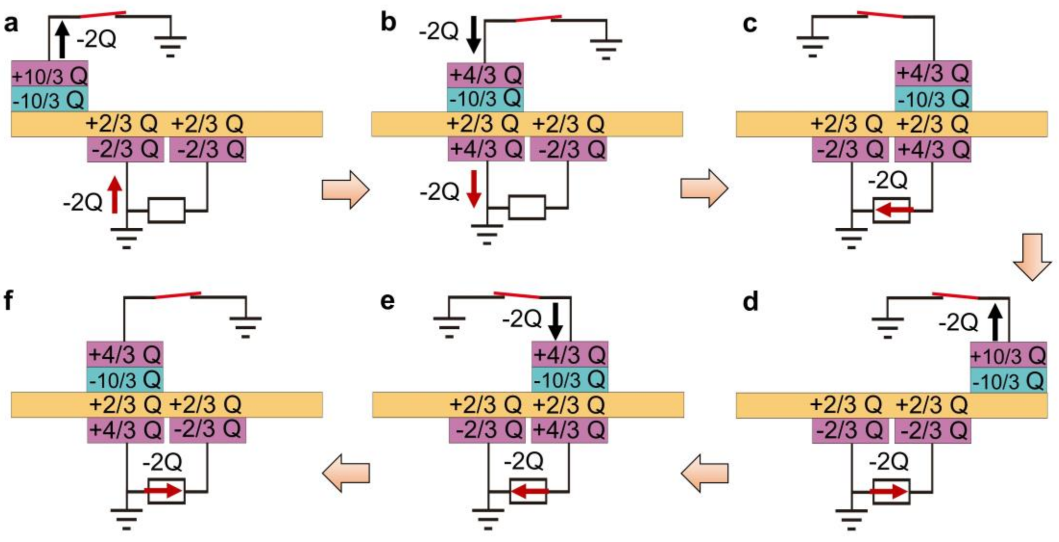

Supplementary Figure 5 | Working mechanism of the CSA-S-TENG. Transferred charges of CSA-S-TENG are twice that of traditional S-TENG.

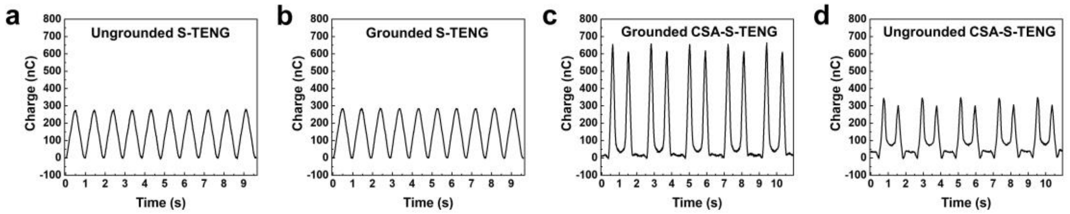

Supplementary Figure 6 | Detailed transferred charge waveform in the four mode of Figure 1j in sequence.

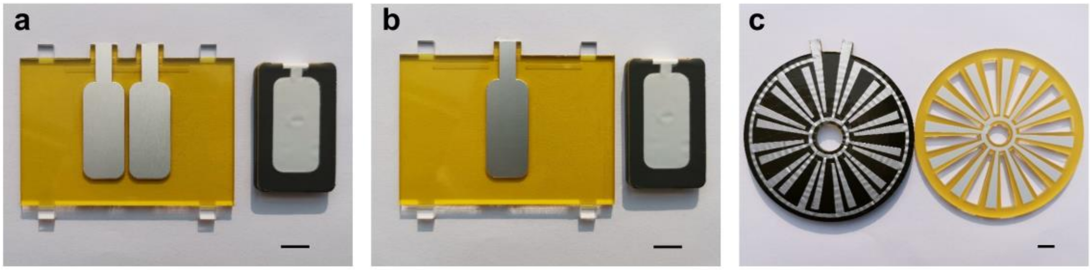

Supplementary Figure 7 | Photographs of three devices in this work. a Photograph of the double-bottom-electrode CSA-S-TENG. **b** Photograph of the single-bottom-electrode CSA-S-TENG. **c** Photograph of the rotation-type CSA-S-TENG. Scale bar, 1 cm.

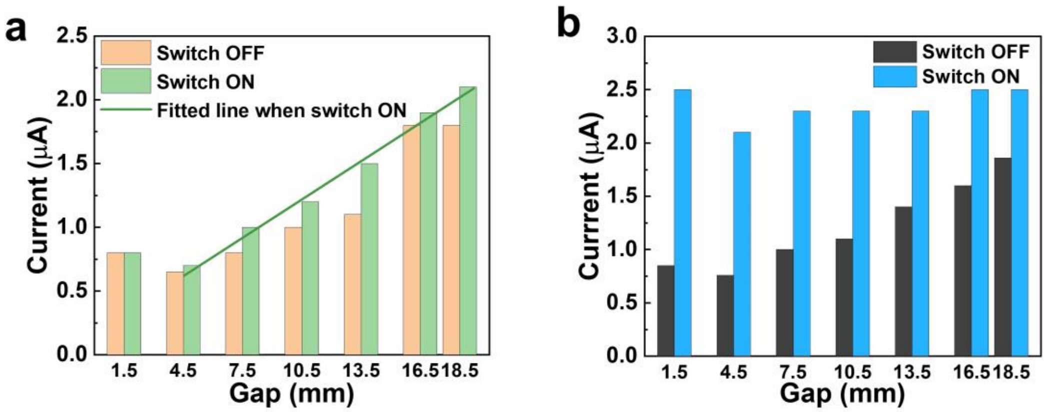

Supplementary Figure 8 | Electrical performance of CSA-S-TENG in different electrode gap. a Short-circuit current of the CSA-S-TENG in different gap when the switch is OFF/ON. Note: Slider just moves on PA film with electrodes coated below it. **b** Short-circuit current of the CSA-S-TENG in different gap when the switch OFF/ON under the condition that slider moves on all PA film.

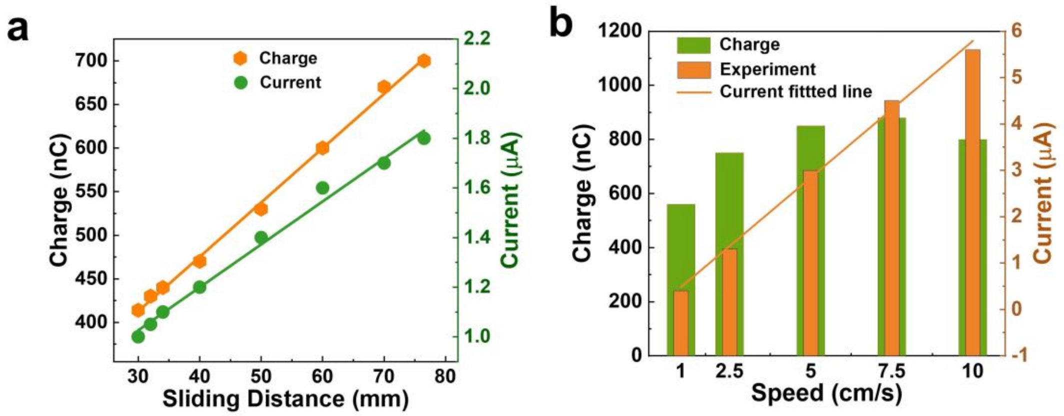

Supplementary Figure 9 | Output performance of single-bottom-electrode CSA-S-TENG under different sliding distances and speeds. a Transferred charges and short-circuit current of the CSA-S-TENG at different sliding distances (fixed sliding speed: 4 cm s^{-1}). **b** Transferred charges and short-circuit current of the CSA-S-TENG at different sliding speeds (fixed sliding distance).

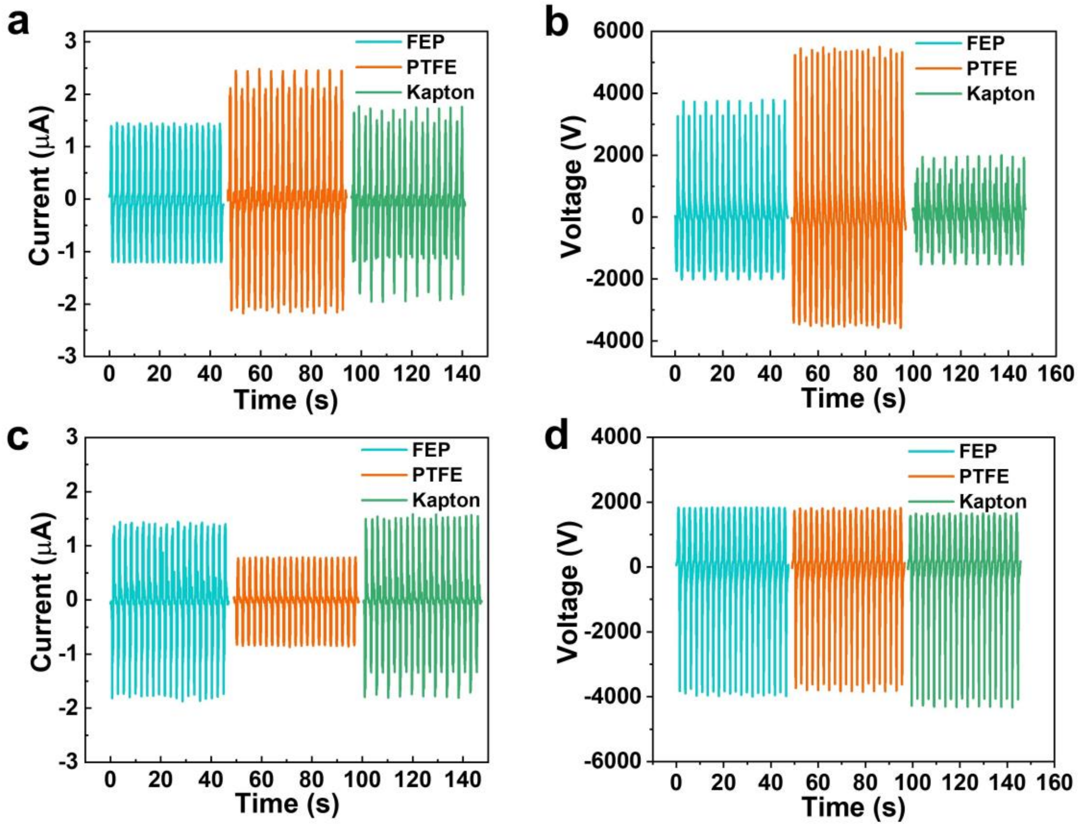

Supplementary Figure 10 | Output performance of single-bottom-electrode CSA-S-TENG with different tribo-materials. **a** Short-circuit current and **b** open-circuit voltage of the CSA-S-TENG when the material of stator is PA and the materials of the slider are FEP, PTFE and Kapton, respectively. **c** Short-circuit current and **d** open-circuit voltage of the CSA-S-TENG when the material of slider is PA and the materials of the stator are FEP, PTFE and Kapton, respectively.

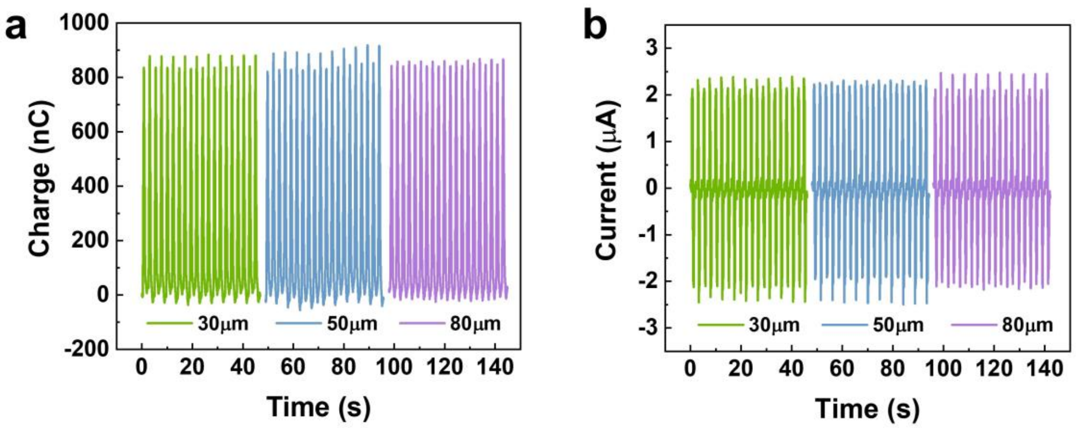

Supplementary Figure 11 | The effect of thickness on top triboelectric layer (PTFE) of slider. a Transferred charges and **b** short-circuit current of single-bottom-electrode CSA-S-TENG at different thicknesses of PTFE (30 μm, 50 μm, 80 μm).

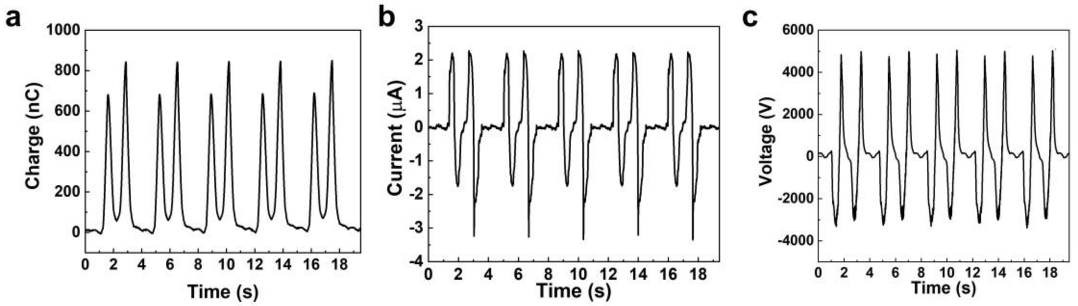

Supplementary Figure 12 | The detailed output waveform of double-bottom-electrode CSA-S-TENG. **a** Transferred charge, **b** short-circuit current and **c** open-circuit voltage of the double-bottom-electrode CSA-S-TENG (Sliding speed: 5 cm s^{-1}).

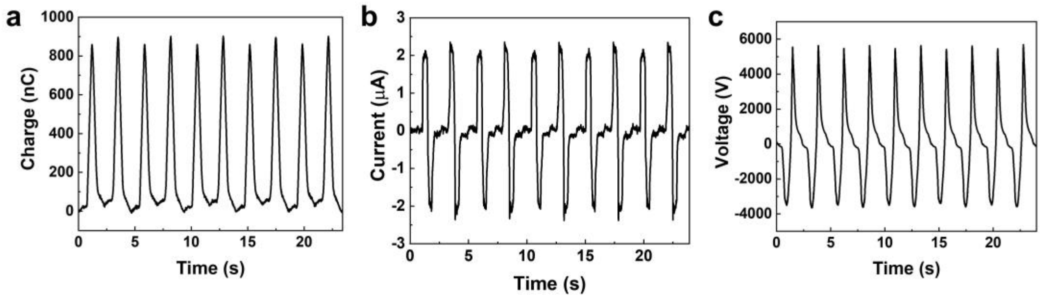

Supplementary Figure 13 | The detailed output waveform of single-bottom-electrode CSA-S-TENG. **a** Transferred charge, **b** short-circuit current and **c** open-circuit voltage of the single-bottom-electrode CSA-S-TENG at sliding velocity of 4 cm s^{-1}.

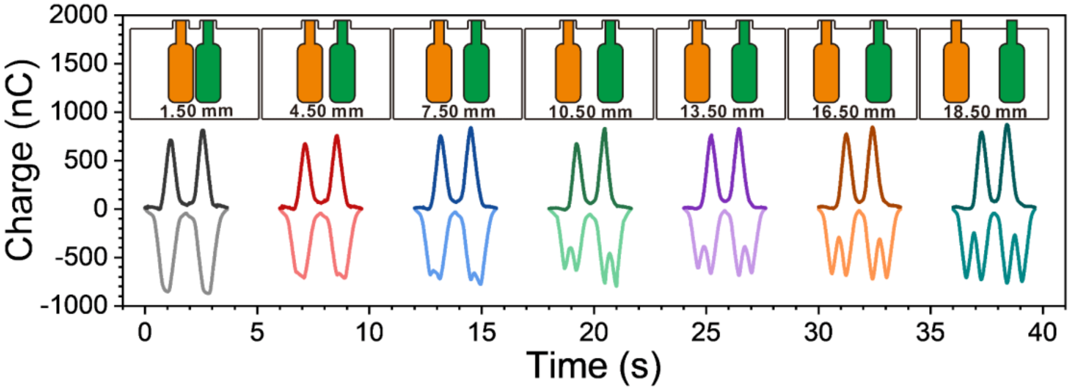

Supplementary Figure 14 | The output waveforms with different electrode gaps. The output channel 1 has a stable waveform (positive). However, the waveform of output channel 2 changes with the increase of gap, because larger gap contributes to increasing blank-tribo-area.

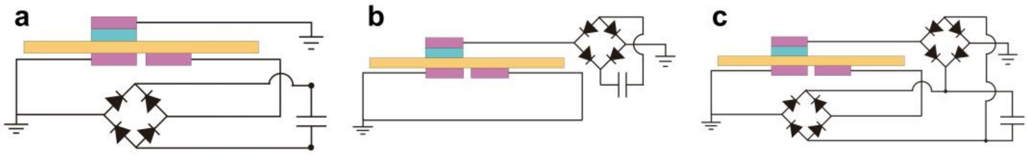

Supplementary Figure 15 | The rectifier circuit diagram of CSA-S-TENG with different channels. a Charging capacitor by output channel 1. **b** Charging capacitor by output channel 2. **c** Charging capacitor with two output channels together.

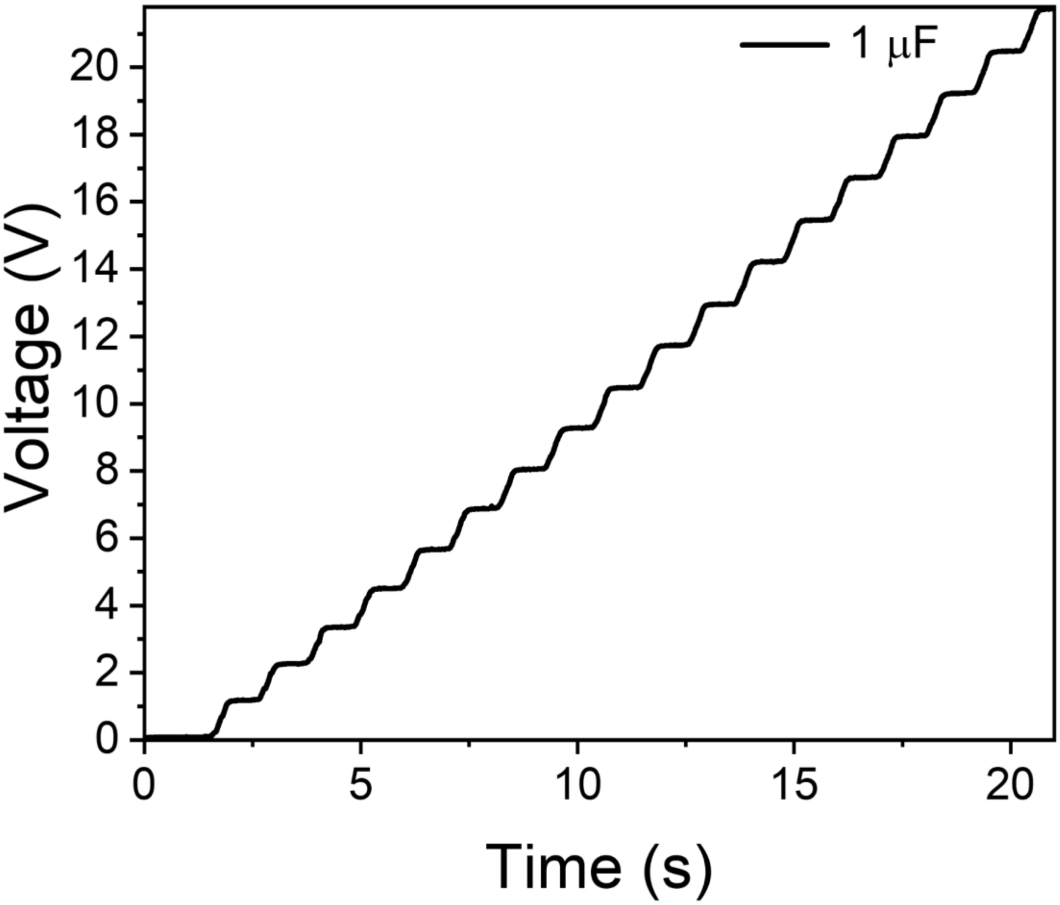

Supplementary Figure 16 | The voltage step of charging capacitor. Charging a 1 μF capacitor to 21.8 V within 20s by CSA-S-TENG at 1 Hz. The big step of the charging voltage indicates the high charge density.

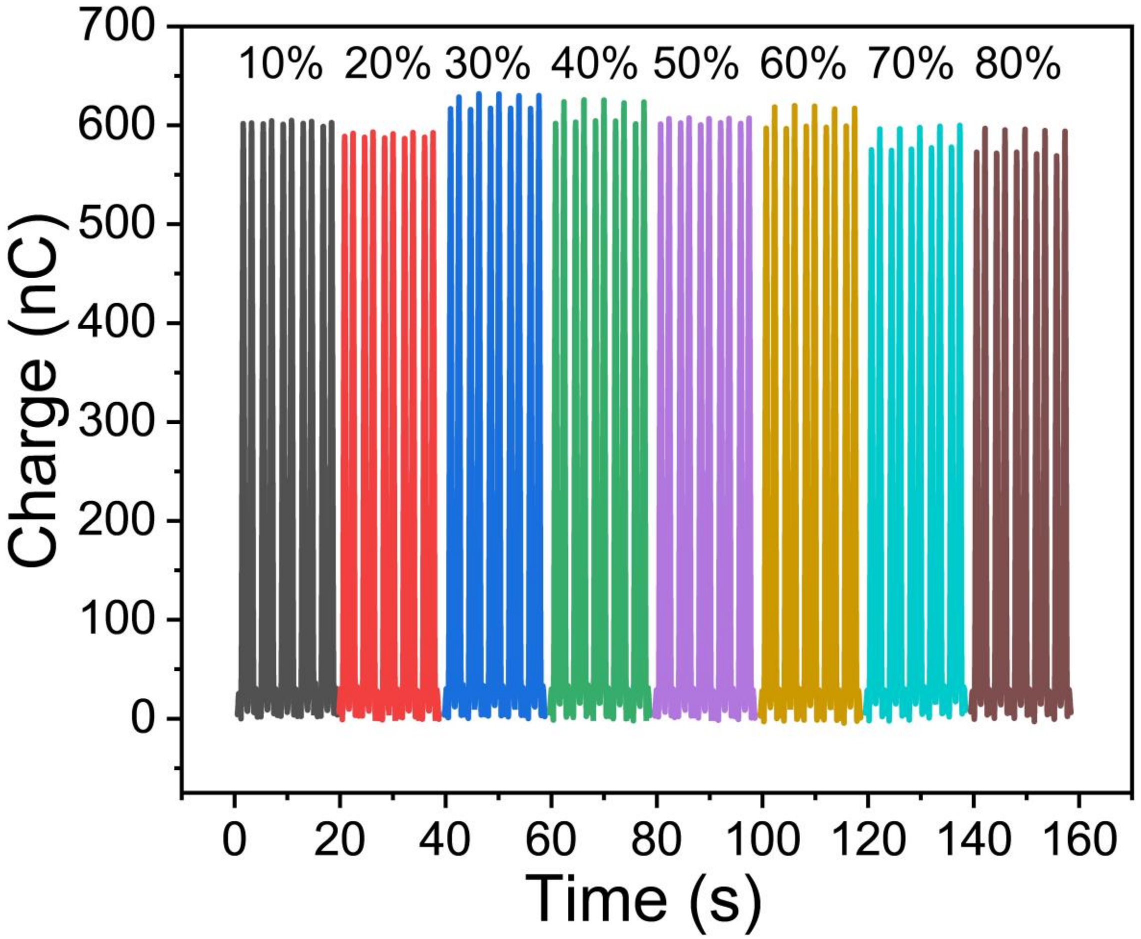

Supplementary Figure 17 | Transferred charges of CSA-S-TENG in different humidity. Due to the characteristics of the S-TENG, the slider is always in close contact with the tribo-layer of stator, so the humidity has little influence on the slider. Since the surface charge dissipation of the extra tribo-layer is beneficial to charge accumulation, humidity has little influence on SCA-S-TENG.

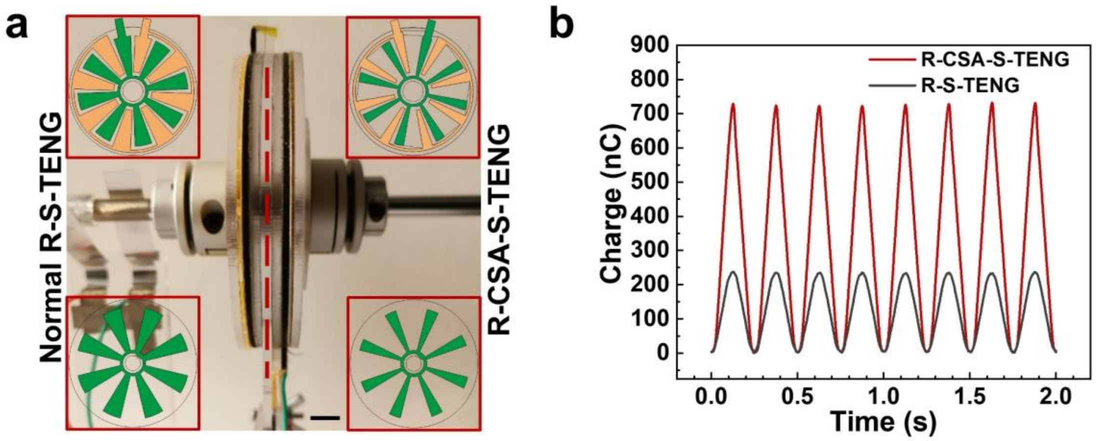

Supplementary Figure 18 | Comparison of R-CSA-S-TENG and normal rotary mode TENG. **a** The photograph of rotary mode CSA-S-TENG and normal rotary mode TENG. Left side disk is normal rotary mode TENG, right side disk is rotary mode CSA-S-TENG. They are both working under the same condition. Scale bar, 1 cm. **b** Output charge of R-CSA-S-TENG and normal rotary mode TENG.

Supplementary Notes

Supplementary Note 1. Charge distribution and calculation of S-TENG with grounded screening electrode.

Firstly, the charge distribution of common sliding TENG under saturation state is shown in Supplementary Figure 1, the saturated surface charge density and effective area of slider are σ and S, respectively, so that the saturated charge of dielectric can be set as Q, where $Q=\sigma\cdot S$. According to the law of charge conservation, there must be equal negative charges generated on PTFE and positive charges generated on nylon (PA) during the triboelectrification process, and the charges distribute in the frictional surface evenly after repeated sliding process. There are +1/2 Q charges on the left bottom electrode (LBE) and -1/2 Q charge on the right bottom electrode (RBE) as the result of electrostatic induction and electrostatic equilibrium (Supplementary Figure 1a, Ⅰ). The transferred charge Q_t of sliding TENG is the difference of charge in two bottom electrodes:

$$Q_t = Q_{\mathrm{LBE}} - Q_{\mathrm{RBE}} \tag{1}$$

And there is a maximum charge -Q transferring from RBE to LBE when slider moves from left side to right (Supplementary Figure 1a, Ⅱ).

Secondly, the top electrode and bottom electrode are both connected to ground (Supplementary Figure 1b, Ⅰ), which means the bottom electrode is connected to the top electrode, and it is equal to two capacitors in parallel as shown in Supplementary Figure 1b, Ⅱ. Here, the voltage of capacitor 1 (V_1) and the voltage of capacitor 2 (V_2) tend to be equal for Point #1 and #2 have the equal potential when the top and bottom electrodes are grounded. And a new charge distribution are formed when the top and bottom electrode are grounded.

$$U_1=U_2 \tag{2}$$

The plate capacitance equation and the relative of charge, voltage and capacitance are:

$$C = \frac{\varepsilon_0 \varepsilon_r S}{d} \tag{3}$$

$$V = \frac{Q}{C} \tag{4}$$

Where C is the capacitance of capacitor in Supplementary Figure 1b, Ⅱ, ε_0 is the

permittivity of vacuum, ε_r is the relative permittivity of dielectric layer, S is the area of electrode, d is thickness of dielectric.

According to Supplementary Equation 2-4, we can get the relative charge in overlapped top (Q_1) and bottom (Q_2) electrodes.

$$\frac{Q_2}{Q_1} = \frac{d_1\varepsilon_{r2}}{d_2\varepsilon_{r1}} \tag{5}$$

The relative charge in left bottom electrode and surface of top tribo-material is:

$$Q_0 = Q_1 + Q_2 + Q_3 \tag{6}$$

Where Q_3 is the charge on the tribo-layer facing the bottom electrode. Q_0 is the charge on the surface of top tribo-layer.

According to Supplementary Equation 5 and 6, the charge in left bottom electrode can be described as follow when electrodes are grounded:

$$Q_2 = \frac{Q_0 - Q_3}{\frac{d_2\varepsilon_{r1}}{d_1\varepsilon_{r2}} + 1} \tag{7}$$

The transferred charge Q_t between the two bottom electrodes when both the top and bottom electrode are connected to ground can be described as:

$$Q_t = Q_{\mathrm{LBE}} - Q_{\mathrm{RBE}} = \frac{Q_0 - Q_3}{\frac{d_2\varepsilon_{r1}}{d_1\varepsilon_{r2}} + 1} - Q_3 \tag{8}$$

Where Q_3 is the charge quantity in the right bottom electrode, and it is equal to the charge quantity on the tribo-layer that faces the right bottom electrode duo to electrostatic induction due to electrostatic induction.

As for the calculation of charge density σ, it can be described by equation below.

$$\sigma = \frac{Q_t}{S} \tag{9}$$

Where S is the effective area of slider, and the definition of S is the same as pervious reports[26,40].

Thirdly, the saturated state charge distributes on the top and bottom tribo-layer that faces the bottom electrode after it is grounded. Because part of charges transfer from left bottom electrode to the grounded shielding electrode under electrostatic equilibrium, the original saturated charge Q state has been broken. Thus, a new charge saturated state of top tribo-layer Q_{0g}, bottom tribo-layer and bottom electrode is obtained again after the top electrode is grounded:

$$Q_{2\mathrm{g}} + Q_{3\mathrm{g}} = Q \tag{10}$$

$$Q_{0g} = 2Q_{3g} \tag{11}$$

$$Q_{0g} = Q_{3g} + Q_{2g} + Q_{1g} \tag{12}$$

$$\frac{Q_{2g}}{Q_{1g}} = \frac{d_1 \varepsilon_{r2}}{d_2 \varepsilon_{r1}} \tag{13}$$

According to the equations above, we can get the new saturated charge of top tribo-layer Q_{0g}, bottom tribo-layer that faces the left bottom electrode Q_{3g} and bottom electrode Q_{2g}.

$$Q_{0g} = \frac{2Q(\frac{d_2 \varepsilon_{r1}}{d_1 \varepsilon_{r2}}+1)}{\frac{d_2 \varepsilon_{r1}}{d_1 \varepsilon_{r2}}+2} \tag{14}$$

$$Q_{3g} = \frac{Q(\frac{d_2 \varepsilon_{r1}}{d_1 \varepsilon_{r2}}+1)}{\frac{d_2 \varepsilon_{r1}}{d_1 \varepsilon_{r2}}+2} \tag{15}$$

$$Q_{2g} = Q - \frac{Q(\frac{d_2 \varepsilon_{r1}}{d_1 \varepsilon_{r2}}+1)}{\frac{d_2 \varepsilon_{r1}}{d_1 \varepsilon_{r2}}+2} \tag{16}$$

Here, we suppose that $d_1/\varepsilon_{r1}= d_2/\varepsilon_{r2}$ and $S_1=S_2$ in order to simplify the calculation, furthermore, this relationship was maintained in our experiment. Thus, the charge density $\sigma_1=\sigma_2$ and the surface charges $Q_1=Q_2$ ($Q=\sigma{\cdot}S$). It will reach a new electrostatic equilibrium when top electrode is grounded and set the surface charges of PTFE as Q_T ($Q_T=Q_{0g}$). The charge distribution shown in Supplementary Figure 1b, Ⅳ is due to electrostatic induction and electrostatic equilibrium. In this occasion, there is 1/4 Q_T shielded by top grounded electrode. Effective surface charge of PTFE still can reach the maximum value Q. According to the Supplementary Equation 14-16, we can know the new surface charges of PTFE is -4/3 Q (Supplementary Figure 1c) and +2/3 Q on PA. There is -Q transferred charge from RBE to LBE when the slider moves from left side to the right. The total transferred charge in every electrode pair always is -Q even though the number of electrode pairs increased in stationary segment (Supplementary Figure 1d). Hence, it cannot improve the output performance of S-TENG by simply grounding the electrode.

Supplementary Note 2. The charge accumulation process of CSA-S-TENG.

Note that the +*Q* charges on the EA (extra blank-tribo-area) have been gone in the process while the slider moving to the opposite direction, and we have demonstrated this in our experiment (Supplementary Figure 2). Supplementary Figure 2a shows the schematic illustration of measurement of the remnant charges, consisting of a metal-dielectric CS-TENG, a composed of top electrode (Al) and a stationary segment of CSA-S-TENG. By measuring the transferred charges of top electrode when this electrode moves up and down in the four areas after CSA-S-TENG regularly worked for a few minutes, we can know the remained charge quantity on the PA surface. In Supplementary Figure 2, the same color refers to the same area. There are few charges on EA, on the contrary, much charges still retains on the middle triboelectric layer with electrodes underneath (Supplementary Figure 2b). To further demonstrate the reason behind that few charge remain on triboelectric layer without electrodes underneath while much charges retain on those with electrodes underneath, we designed another two kinds of metal-dielectric contact-separated TENG, that are single electrode mode and double electrodes mode (Supplementary Figure 3). Supplementary Figure 3a, c show the charge density for a single electrode mode contact-separated TENG with 50 μm PA and PTFE thin films. Sufficient triboelectrification was applied to the surface of the PA and PTFE film before measurement. Therefore, the initial surface charge density should be the same for the both modes CS-TENG. There is a period of time (about 1-2 s) between the experimental conditions being ready and the beginning of the measurement due to the practical operation of the experiment, and during which time the charge dissipates into the air too. Therefore, it is hard to know its initial charge surface density of PA, but according to Supplementary Figure 3b, the value is at least 50 μC m^{-2}. But in the first time we measure Al-PA single-electrode CS-TENG, the surface charge density is 17 μC m^{-2}, suggesting that the surface charge had dissipated by at least 66% (33 μC m^{-2}). The charge density of double electrode mode CS-TENG are four times higher than that of single electrode mode. This demonstrates that charges are more easily dissipated into air when there are no electrodes under triboelectric layer, while the charges can keep on that with electrodes underneath due to the shielding function of electrode. In other words, the charges on triboelectric layer are bounded by the charges on electrode.

The top electrode and bottom electrode have the equal potential because they are grounded. Supplementary Figure 4 shows the process of triboelectrification and electrostatic induction of CSA-S-TENG. When the slider starts from the left to approach EA, all charges (-4/3 Q) of top tribo-layer and charges induced before are shielded by top grounded electrode again (Supplementary Figure 4a). As mentioned above, the effective surface charge of PTFE still can reach the maximum value Q. Hence, there are -7/3 Q negative charges on PTFE and +Q positive charges on PA due to triboelectrification (Supplementary Figure 4b). When slider moves from RBE to LBE, there are +5/6 Q charges on both top electrode and LBE as a result of electrostatic equilibrium (Supplementary Figure 4c). The transferred charges increase to -3/2 Q when the slider moves to RBE (Supplementary Figure 4d). The slider continues to move right to EA, it will increase +Q charges on PA and -Q on PTFE again, based on the same principle (Supplementary Figure 4e, f). The transferred charges increase to -2 Q when the slider move to the LBE (Supplementary Figure 4g-i).

Supplementary Note 3. The charge transfer process and mechanism of CSA-S-TENG.

Supplementary Figure 5 shows the working mechanism of CSA-S-TENG. The surface charges of PTFE reach the limited maximum value after several cycles. And there are few charges on EA due to the balance between charge induction and dissipation. There are -10/3 Q charges on PTFE, +10/3 Q on top electrode, -2/3 Q on LBE and +2/3 Q on the corresponding triboelectric layer (Supplementary Figure 5a). -2 Q charges transfer from ground to top electrode when slider moves on LBE (Supplementary Figure 5b) and -2 Q charges transfer from RBE to LBE when it is on RBE (Supplementary Figure 5c). When it moves to the far right, -2 Q charges transfer from top electrode to ground while equal charges transfer to RBE (Supplementary Figure 5d). Then the slider moves backward to finish the second half cycle. 2 Q charges are transferred per cycle due to the ultrahigh surface charge density of PTFE. Therefore, we demonstrate the charge density of CSA-S-TENG is as twice as that of the normal S-TENG theoretically.

To further demonstrate the improvement in experiment, Figure 1J shows the transferred charges of the four sliding modes of TENG under the same condition, including common sliding TENG (S-TENG), sliding TENG with grounded top screening electrode (grounded S-TENG), CSA-S-TENG and S-TENG which is just with EA (ungrounded CSA-S-TENG). This is realized by controlling the linear motor and the switch (Supplementary Movie 1). There is an obvious improvement of effective transferred charges of CSA-S-TENG. The transferred charges (Q_{SC}) of grounded S-TENG at short-circuit is 287 nC, which has no big difference compared with 280 nC of S-TENG. As the slider moves away from the EA, the output gradually becomes stable after several cycle. Q_{SC} of CSA-S-TENG stabilizes at 666 nC at last, which is 2.3 times higher than that of normal S-TENG, more details are shown in Supplementary Figure 6. After that, turn off the grounded switch at top electrode, the Q_{SC} of ungrounded CSA-S-TENG decreases sharply and stays around 347 nC finally. The whole process demonstrates that both the extra blank-tribo-area and screening electrode are two extremely important parts for the high surface charge density of CSA-S-TENG.
