# Supplementary material for: Boosting output performance of sliding mode triboelectric nanogenerator by charge space-accumulation effect
Source: Nat Commun. 2020 Aug 26;11:4277. doi: 10.1038/s41467-020-18086-4 (PMC7450049; doi:10.1038/s41467-020-18086-4)
Supplement: Supplementary file 3 — Description of Additional Supplementary Files [file 41467_2020_18086_MOESM3_ESM.pdf]

Description of Additional Supplementary Files

File Name: Supplementary Movie 1
Description: Both shielding electrode and extra blank-tribo-area are necessary for CSA-S-TENG.

File Name: Supplementary Movie 2
Description: Demonstration of the stepwise charging voltage of 1μF capacitor.

File Name: Supplementary Movie 3
Description: 912 green LEDs are powered by R-CSA-S-TENG

File Name: Supplementary Movie 4
Description: A digital scientific calculator is powered by R-CSA-S-TENG.

File Name: Supplementary Movie 5
Description: A commercial multifunctional thermo-hygrometer is powered by R-CSA-S-TENG.
